# Efficient Eucalypt Cell Wall Deconstruction and Conversion for Sustainable Lignocellulosic Biofuels

**DOI:** 10.3389/fbioe.2015.00190

**Published:** 2015-11-20

**Authors:** Adam L. Healey, David J. Lee, Agnelo Furtado, Blake A. Simmons, Robert J. Henry

**Affiliations:** ^1^Queensland Alliance for Agriculture and Food Innovation, University of Queensland, St. Lucia, QLD, Australia; ^2^Forest Industries Research Centre, University of the Sunshine Coast, Maroochydore, QLD, Australia; ^3^Department of Agriculture and Fisheries, Forestry and Biosciences, Agri-Science Queensland, Gympie, QLD, Australia; ^4^Joint BioEnergy Institute, Lawrence Berkeley National Laboratory, Emeryville, CA, USA; ^5^Biological and Engineering Sciences Center, Sandia National Laboratories, Livermore, CA, USA

**Keywords:** eucalypts, biotechnology, pretreatment, lignocellulosic biofuel, bioenergy

## Abstract

In order to meet the world’s growing energy demand and reduce the impact of greenhouse gas emissions resulting from fossil fuel combustion, renewable plant-based feedstocks for biofuel production must be considered. The first-generation biofuels, derived from starches of edible feedstocks, such as corn, create competition between food and fuel resources, both for the crop itself and the land on which it is grown. As such, biofuel synthesized from non-edible plant biomass (lignocellulose) generated on marginal agricultural land will help to alleviate this competition. Eucalypts, the broadly defined taxa encompassing over 900 species of *Eucalyptus*, *Corymbia*, and *Angophora* are the most widely planted hardwood tree in the world, harvested mainly for timber, pulp and paper, and biomaterial products. More recently, due to their exceptional growth rate and amenability to grow under a wide range of environmental conditions, eucalypts are a leading option for the development of a sustainable lignocellulosic biofuels. However, efficient conversion of woody biomass into fermentable monomeric sugars is largely dependent on pretreatment of the cell wall, whose formation and complexity lend itself toward natural recalcitrance against its efficient deconstruction. A greater understanding of this complexity within the context of various pretreatments will allow the design of new and effective deconstruction processes for bioenergy production. In this review, we present the various pretreatment options for eucalypts, including research into understanding structure and formation of the eucalypt cell wall.

## Introduction

Currently, approximately 40% of the world’s transportation fuels (fossil fuels) are derived from non-renewable sources, the combustion of which directly contributes to global climate change (Simmons et al., 2008; González-García et al., 2012). As such, renewable plant-based feedstocks for fuel synthesis, aptly referred to as “biofuels,” are under consideration to alleviate these concerns. The first generation of feedstocks used for biofuel synthesis was mainly derived from sugarcane and corn, as their energy storage polysaccharides are readily available and easily hydrolyzed into monosaccharides for microbial fermentation. However, as these feedstocks are important links within the human food chain, generation of biofuel from these crops creates a direct competition for resources. Furthermore, in the USA alone, the maximum biofuel yield from the first-generation biofuel feedstocks is roughly 30% of the renewable fuel target (Perlack et al., 2005), creating a large gap that must be filled with alternatives. Plant cell wall structural polysaccharides, although more complex than starch molecules, represent the most abundant biopolymers in the world, containing large stores of carbon for conversion into liquid fuels, such as ethanol and butanol (Wyman, 1999). As structural polysaccharides represent the non-edible portions of plants, fuel synthesized from cellulose and hemicellulose can help alleviate the competition between energy and agriculture. Crops intended for this purpose are known as the second-generation biofuel feedstocks.

There are numerous advantages to using the second-generation feedstocks as a source of renewable energy. Combustion of fossil fuels adds carbon dioxide to the atmosphere, the main contributor to the greenhouse effect and subsequent climate change. Biofuel crops help to mitigate the effect of CO_2_ by sequestering more carbon within their biomass than is released during biofuel combustion, thus creating a net reduction in CO_2_ levels (Rubin, 2008; Shepherd et al., 2011; Soccol et al., 2011). High production grassy species, such as those belonging to the *Miscanthus* and *Saccharum* genera, are high-value bioenergy crops due to their exceptional growth rate and desirable biomass composition that is relatively easy to deconstruct for polysaccharides using mild pretreatments (Rubin, 2008). However, high production crops, such as these require nutrient rich soils, normally reserved for intensive agriculture. This indirect competition for land and soil between food or fuel crops can be avoided through the cultivation of feedstocks that grow well on marginal land, of which there is approximately 1.4 billion hectares available globally (Carroll and Somerville, 2009; Somerville et al., 2010). Fuel production from woody (or lignocellulosic) biomass also offers several advantages over grassy biomass. Growing trees for energy production allows biomass to be “stored on the stump” to be harvested when needed (Shepherd et al., 2011), a luxury not afforded by grasses, which must be harvested at particular times during the year and must be processed immediately before fungal degradation begins. Also, woody biomass can be transported to processing facilities more economically, as it more energy dense than grassy biomass which requires greater amounts of fuel to move the biomass than can be generated from its fibers (Kaylen et al., 2000; Somerville et al., 2010).

Eucalypts, a native Australian taxon that includes genera *Eucalyptus*, *Corymbia*, and *Angophora*, are an attractive prospective biofuel crop, being the most widely planted hardwood trees in the world (Myburg et al., 2007; Grattapaglia and Kirst, 2008). Having adapted to the terrestrial environment of Australia, eucalypts are well suited for plantations in a wide variety of climates, soil types, and rainfall conditions (Ladiges et al., 2003; Myburg et al., 2007; Grattapaglia and Kirst, 2008). They are grown commercially in over 100 countries with well-established silviculture practices already in place, such as clonal propagation, allowing plantations to achieve high rates of productivity, up to 25 dry tonnes/hectare/year (Stricker et al., 2000; Rockwood et al., 2008), more than double the required productivity rate estimated by the US Department of Energy for a long-term renewable energy crop (Hinchee et al., 2009). Furthermore, many eucalypt species also regenerate shoots after harvesting, which ensures ease of management by potentially eliminating the need for re-planting (Shepherd et al., 2011).

Eucalypts, due to differences in flowering times (protantry) and self-incompatibility, are predominately out-crossing species which maintains high levels of heterozygosity in their genomes and encourages genetic diversity and phenotypic variation (Horsley and Johnson, 2007; Grattapaglia and Kirst, 2008). This variation is exploited by breeders through selection and combination of desirable traits for industrial application, such as controlled-cross hybrids that combine the high cellulose and fiber content of *Eucalyptus globulus* with the growth rate and form of *E. grandis* (Poke et al., 2005; Grattapaglia, 2008). Phenotypic traits that are desirable for efficient biofuel production are closely aligned with those sought by the pulp and paper industry. High-quality wood pulp is primarily composed of cellulosic fibers, which upon enzymatic hydrolysis releases monomeric glucose subunits, which serve as the main substrate for microbial fermentation and conversion to liquid fuel (Hisano et al., 2009; Wegrzyn et al., 2010).

Despite the advantages of lignocellulosic biofuel crops, woody biomass conversion into a source of renewable energy in hindered through its natural complexity and recalcitrance to deconstruction (Ramos and Saddler, 1994; Blanch et al., 2011). Many of the options for deconstruction require harsh and expensive chemicals (such as acids and alkalis) or energy intensive methods, such as grinding and ball milling. An increased understanding of eucalypt biomass will allow the engineering of more cost-effective pretreatments that can increase fuel production efficiency, while lessening the formation and impact of inhibitory compounds produced during conversion. In this review, we present an overview of the major contributing components of eucalypt cell wall recalcitrance, and the current research surrounding eucalypt biomass pretreatment for fuel production.

## Challenges to Lignocellulosic Biofuel Conversion

**Figure 1:**
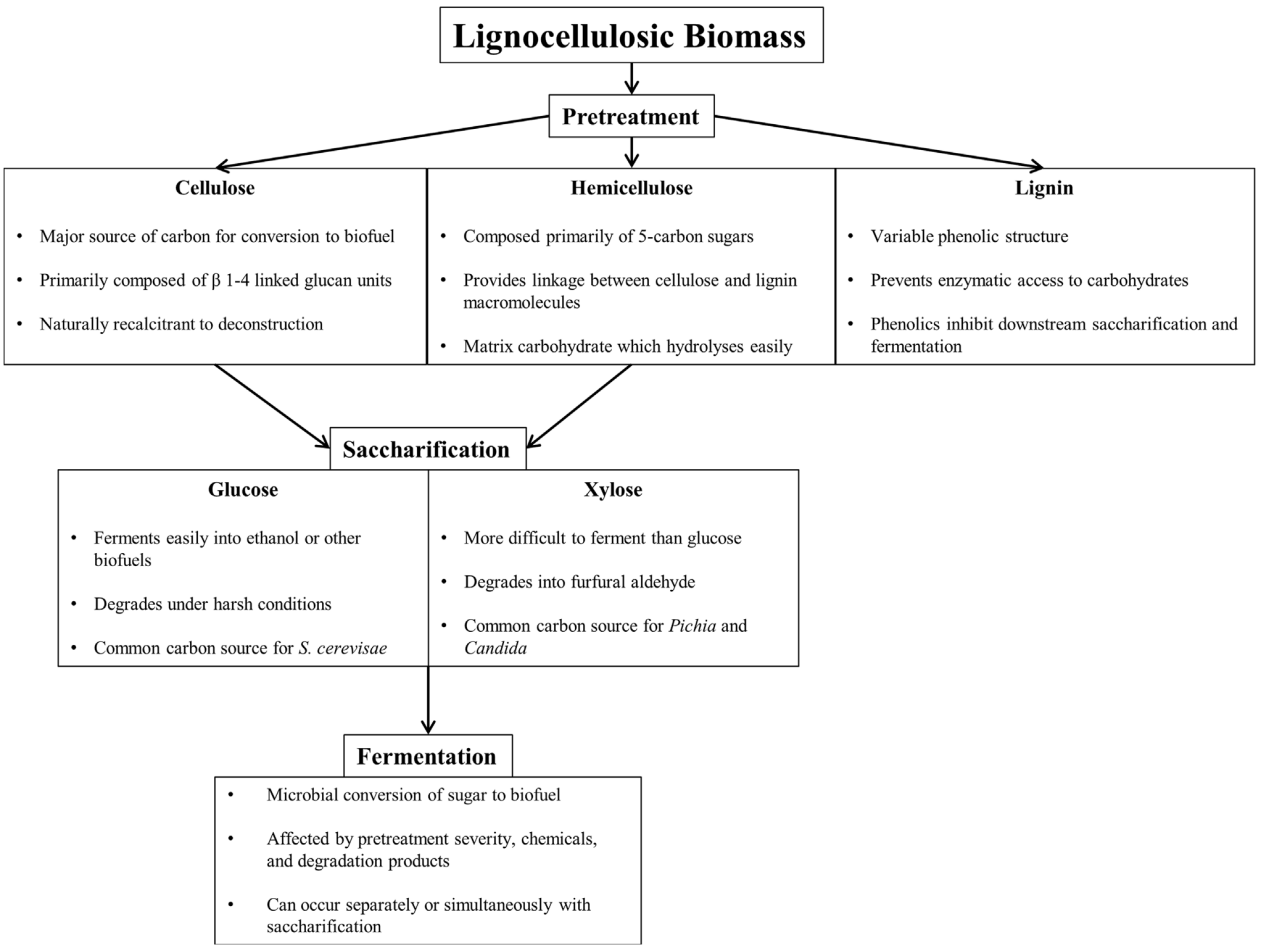
**Component overview of lignocellulosic deconstruction, saccharification, and fermentation for biofuel production**.

Efficient conversion of lignocellulosic biomass to biofuel requires pretreatment, saccharification, and fermentation, each presenting unique challenges (Figure 1). Pretreatment breaks down and separates each of the major components of biomass (cellulose, hemicellulose, and lignin), either through mechanical or through chemical means to reduce cellulose crystallinity, increase surface area, and remove lignin, the largest barrier to efficient enzymatic saccharification (Furtado et al., 2014). Table 1 summarizes common lignocellulose pretreatments and highlights pros and cons of each process.

**Table 1:** **Summary and assessment of common pretreatment options for lignocellulose**.

Pretreatment	Summary	Pros	Cons
Grinding and milling	Mechanical disruption of biomass to increase surface area	No chemicals required	Energy inefficient
		No degradation products generated	Lignin structure remains
Concentrated acid	Relatively complete hydrolysis of biomass with hydrochloric or sulfuric acid	Complete biomass hydrolysis	High cost and loss of acid
		Low inhibitory product formation under low temperature conditions	High environmental impact
			Phenolic release
			Inhibition of fermentation
Dilute acid	Combination of acid and high temperature to solubilize hemicellulose	Low acid concentrations required (<1%)	Sugar degradation and loss
		Short reaction times	Release of phenolics
Alkaline	Cleaves linkages within lignin and between hemicellulose and lignin	Swells biomass	High environmental impact
		Established pulping practice	Low recovery
		Works with various feedstocks	Requires neutralization
		Low temperature, low pressure reaction	
Organosolv	Aqueous/organic solvent at high temperatures break hemicellulose–lignin bonds	Allows intact lignin recovery	Organic solvents are expensive and inhibit fermentation
		Works well across various feedstocks	High temperatures (250°C) required
Steam explosion	Biomass explosion of biomass by high temperature/pressure coupled with rapid decompression	Solubilization of hemicellulose and reduced cellulose crystallinity	High temperatures generate inhibitory products
		Short reaction time	
Autohydrolysis	Pressurized, high temperature water solubilizes hemicellulose with *in situ* acids	No chemicals needed	Requires a low lignin feedstock to be efficient
		Low environmental impact	High temperature and pressure required
Ionic liquids	Room-temperature organic liquid salts dissolve biomass	Selective precipitation of cellulose	High cost of chemicals
		Lignin recovery	Inhibition of microbial fermentation
		Stable, low volatility chemicals	
		Works well regardless of varying wood properties	

*Hendriks and Zeeman (2009), Alvira et al. (2010), and Blanch et al. (2011)*.

Despite the low cost of producing lignocellulosic biomass, the economic cost of producing biofuel remains high (Lange, 2007). Pretreatment, a required process for increasing saccharification is costly, requiring large amounts of energy or expensive chemicals (e.g., sulfuric acid) to promote enzymatic access to polysaccharides. Fermentation also represents a significant cost to biofuel production as the production of enzymes is expensive, and the efficiency at which microorganisms can convert sugars into fuel is dependent on pretreatment (Hamelinck et al., 2005). Therefore, harsh pretreatments that are used for biomass deconstruction in other industrial processes (e.g., pulping) may not be appropriate for biofuel production. There are also significant operational costs associated with biofuel conversion, including capital costs, labor, and waste water processing. As such, the development of simple, cost-effective, and environmentally safe pretreatments is critical for large-scale sustainable production. Given that pretreatment and fermentation represent the highest costs of producing biofuel, feedstock selection is also critical for fuel production as well.

The simplest pretreatment option is grinding and milling of biomass to increase reactive surface area for hydrolysis. However, the energy required to generate small enough particles is often too high to be a cost-effective option (Zheng et al., 2009; Talebnia et al., 2010). A more common pretreatment is acid hydrolysis, where strong acids (e.g., H_2_SO_4_) solubilize the hemicellulose polysaccharide matrix, leaving behind cellulose and lignin (Galbe and Zacchi, 2007). Although effective, acid pretreatment generates compounds that inhibit downstream biomass conversion processes through reduction of microbial growth and enzymatic release (Jönsson et al., 2013). For instance, while the majority of lignin present in the cell wall is acid insoluble (Klason lignin), upon pretreatment, a small portion hydrolyzes releasing phenolics, such as vanillin, trans-cinnamic acid, and 4-hydrobenzoic acid (Palmqvist and Hahn-Hägerdal, 2000; Ximenes et al., 2010). Additionally, monomeric subunits of cellulose and hemicellulose degrade in low pH conditions, generating aldehydes [furfural and 5-hydroxymethyl-2-furaldehyde (5-HMF)] and organic acids. Formation of these degradation products inhibits fermentation by reducing available sugars and limiting microbial growth (Zheng et al., 2009; Soccol et al., 2011; Puri et al., 2012). Similarly, organosolv pretreatment combines an organic solvent (e.g., ethanol) with an inorganic acid catalyst (e.g., sulfuric acid) to destroy internal lignin and hemicellulose bonds, resulting in effective recovery of high-quality cellulose and lignin portions of biomass. Although an effective pretreatment for both hardwood and softwood biomass, downstream ethanol production still ­suffers from the formation of inhibitory products (Sun and Cheng, 2002; Zhu and Pan, 2010).

Alkaline pretreatment, which employs chemicals, such as sodium hydroxide, lime and hydrazine, to disrupt the linkage between hemicellulose and lignin, reduces the formation of inhibitory products but nonetheless remains an expensive option that is dependent on lignin content which determines its efficacy (Blanch et al., 2011). An alternative method, which seeks to work universally well regardless of biomass composition, is ionic liquid (IL) pretreatment. ILs are non-volatile, stable compounds that solubilize lignocellulosic biomass, allowing selective precipitation of components for easy recovery. Once dissolved, cellulose precipitates from solution upon addition of an antisolvent (e.g., water or ethanol) while lignin and other solutes remain intact (Zhu et al., 2006; Singh et al., 2009).

## Cellulose Crystallinity

Cellulose, the most abundant biopolymer on earth, is composed of thousands of glucose monomers linked together by β 1–4 glycosidic bonds. Its function within the cell wall is to provide strength and rigidity, while remaining flexible during cell expansion and growth (Mutwil et al., 2008; Mansfield, 2009). Sucrose, generated through photosynthesis, supplies the glucose molecule required for cellulose synthesis, which is phosphorylated by hexokinase, and is incorporated into growing cellulose microfibrils by cellulose synthase (CESA) enzymes (Somerville, 2006; Joshi and Mansfield, 2007; Mohnen et al., 2008). During synthesis, each cellulose microfibril associates with other glucan chain through extensive hydrogen bonding and Van der Waals forces, creating a highly compact polysaccharide. Within the cell wall, cellulose exists in primarily two forms, a highly ordered crystalline structure that lacks surface area and a less ordered, amorphous type (Harris and DeBolt, 2010). The highly compact crystalline structure lends itself toward the natural recalcitrance of woody biomass to deconstruction, as it prevents cellulase enzymes to accessing microfibrils, thus inhibiting efficient saccharification (Mosier et al., 2005; Hall et al., 2010).

Crystalline cellulose formation in eucalypts has been traditionally researched through the formation of tension wood. Tension wood, characterized by the formation a gelatinous layer of crystalline cellulose (G-layer), serves to re-direct a growing stem upwards in response to gravitational stress (Jourez et al., 2001). As tension wood can be artificially induced, Paux et al. (2005) investigated tension wood formation in *E. globulus* by tying the growing stems of 2-year-old trees to the adjacent tree, bending their trunks to a 45° angle. By extracting RNA from the xylem of the bent trees on either side of the bend (tension wood and opposite wood) at various timepoints (0, 6, 24, and 168 h), the authors were able to identify differentially expressed genes during cellulose formation using a xylem complementary DNA (cDNA) array. As evidenced by a much larger bent-stem experiment performed in *Eucalyptus nitens* with 4,900 xylem cDNAs, Qiu et al. (2008) found tension wood, although lacking the characteristic “G-layer,” contained high concentrations of cellulose and low amounts of Klason lignin. Additionally, X-ray diffraction of upper and lower bent stems revealed that the cellulose microfibril angle (MFA) on the upper branch was much less than that of the lower branch. MFA, the angle at which cellulose polymers at synthesized within the cell wall affects their tendency to form hydrogen bonds. MFA, which affects wood stiffness (Schimleck et al., 2001), is an indirect biofuel trait as cellulose content negatively correlates with MFA and lignin content (Plomion et al., 2001). Qiu et al. (2008) also found that in tension wood, the highest expression profiles belonged to β-tubulin genes and fasciclin-like arabinogalactan (FLA) proteins. β-Tubulin proteins are responsible for transporting cellulose synthesis machinery to the plasma membrane, which may in-turn affect MFA. FLA genes, known to associate pectic side-chains and other structural polysaccharides also affect MFA, as demonstrated through transformation of *E. nitens* with FLA3, identified from the *E. grandis* genome (Macmillan et al., 2015).

To investigate the effect of tension and opposite wood on saccharification and fermentation, Muñoz et al. (2011) treated *E. globulus* biomass to organosolv (ethanol/water) pretreatment, followed by simultaneous saccharification and fermentation (SSF) (discussed later). The authors found that tension wood (as compared to opposite wood) contained similar glucan content (46–47%), higher xylan amounts (16.0 and 12.0%, respectively), and lower lignin content (22.1 and 26.1%, respectively). Upon pretreatment, remaining residual lignin was lower in tension wood and required less time and cooking (as expressed by H factor, a single variable calculated from the combination of cooking temperature and time) for delignification. Pulp from tension and opposite wood were assayed for glucose conversion by enzymatic hydrolysis, finding that despite similar or higher lignin content, glucan to glucose conversion was more efficient in opposite wood. However, investigation into pulp viscosity showed that tension wood glucans were of higher molecular mass, which may have influenced their rate of conversion. Upon submission of pulps from tension and opposite wood for SSF, the authors found that harsh pretreatment conditions (H factor – 12,500) outperformed milder conditions (H factor – 3,900) to produce 35 and 30 g/L of ethanol, respectively. Considering the maximum theoretical conversion of ethanol from glucose is 51%, these concentrations represent 95 and 85% conversion efficiency, which scales to a yield of 290 L of ethanol/tonne of biomass. Considering the formation of tension wood is undesirable from a timber standpoint and good management practices within plantations dictate that trees of low economic value are removed to increase the growth of high-value trees (McIntosh et al., 2012), ethanol production from eucalypt plantation thinnings is a potential option for bioenergy production, dependent on distance required for biomass transport, growth rate, and stocking rate.

## Non-Cellulosic Polysaccharides

Before the formation of the secondary cell wall, the plant primary cell wall is a thin yet flexible structure that resists gravity and internal pressure while allowing growth and expansion (Cosgrove, 2005). Cellulose, being the core of the internal structure, provides the scaffold that non-cellulosic polysaccharides, such as hemicellulose and pectin, surround within a polysaccharide matrix (Carpita and Gibeaut, 1993; Mellerowicz and Sundberg, 2008). Although hemicellulose and pectin are polysaccharides, and thus can hydrolyze into monomeric subunits, these monomers consist mainly of pentose sugars which are more difficult to ferment than glucose. As such, based on their difficulty to ferment and how they reduce access to cellulose, hemicellulose and pectin also contribute to biomass recalcitrance (Himmel et al., 2007; Sticklen, 2008).

Xyloglucan is the most abundant hemicellulose polysaccharide of woody dicot species, with a repeating structure of β 1–4 glucan residues with various side-chains, predominantly unbranched glycosyl residues or α 1–6 xylose. Other side-chain molecules include galactose, fructose, and arabinose (Harris and DeBolt, 2010; Scheller and Ulvskov, 2010). Xyloglucan interacts with cellulose by crosslinking with non-crystalline regions or through hydrogen bonding with the microfibrils themselves (Cosgrove, 2005). For further reinforcement and strength, woody plant cell walls synthesize a secondary cell wall of cellulose, hemicellulose, and lignin. However, unlike the primary cell wall with a repetitive hemicellulose structure, the secondary cell wall polysaccharide matrix is composed of highly variable xylan molecules. This varied structure is highly substituted, with the most common modification in woody dicots being glucuronosyl residues which generates glucuronoxylan (Li et al., 2006; Scheller and Ulvskov, 2010). Given that *E. globulus* is a major source of fiber for the pulp and paper industry, the structure of its non-cellulosic polysaccharides has been extensively researched. Originally, eucalypts were believed to possess glucuronoxylan as found in woody dicot species, but investigations by Shatalov et al. (1999) and Evtuguin et al. (2003) found that *E. globulus* xylan structure was highly substituted by galactosyl and acetyl residues. These residues, although not targets for saccharification, can affect downstream conversion efficiency. Galactose is one of the most difficult sugars to ferment (Lee et al., 2011), while acetyl groups can contribute acetic acid during fermentation conditions which inhibits ethanol production in *Pichia* (Ferrari et al., 1992) and *Saccharomyces* (Taherzadeh and Karimi, 2007).

Acid pretreatment, designed to hydrolyze the hemicellulose matrix surrounding cellulose, requires various acid concentrations, pretreatment times, and temperatures to be effective. To examine these parameters on various eucalypt species, McIntosh et al. (2012) conducted a 3^3^ factorial design (acid concentration, temperature, and pretreatment time) to understand sugar solubilization and degradation, enzymatic saccharification in response to pretreatment, and the fermentation of various hydrolyzates. Thinned trees of *Eucalyptus dunnii* and *Corymbia citriodora* subsp. *variegata* at ages 6 and 10 were tested within the factorial design, finding their biomass composition contained approximately 47–48% glucan, 16–17% xylan, 5% minor sugars, and 30% lignin. The authors found that under the mild pretreatment conditions [expressed as a combined severity factor (CSF)], monomeric xylose was the first to solubilize. However, as pretreatment became more severe, recovered xylose yields decreased, likely lost to degradation. Glucose release correlated with CSF increase, with temperature being the main contributing factor, followed by acid concentration and reaction time. In the presence of crude *E. dunnii* hydrolyzate, *Saccharomyces cerevisiae* could be cultured for fermentation, although the time (30 h) at which the organism was able to convert 38 g of glucose into 18 g/L of ethanol (92% efficiency) was double when compared to starch-fed fermentations (Sánchez and Cardona, 2008). This study highlights the cost/benefit analysis of biomass conversion, where more severe treatments will result in greater glucose yields but will generate more degradation products from matrix polysaccharides that inhibit fermentation. The authors also encountered significant differences in saccharification yield between biomass of different ages. After two pretreatment severity conditions (CSF 1.60 and 2.48), 6-year-old eucalypt biomass yielded greater amounts of glucose than their 10-year-old counterparts, despite similar chemical composition. These differences were attributed to changes in cellulose crystallinity, which may be species specific based on similar studies in *Populus* (DeMartini and Wyman, 2011).

Although xylose, the main monosaccharide present within hemicellulose, is more difficult to ferment by fungi due to an overproduction of nicotinamide adenine dinucleotide (NADH) under anaerobic conditions (Bruinenberg et al., 1983), hemicellulose exists as a matrix polysaccharide and is thus far less resistant to pretreatment than cellulose. To demonstrate the ease at which xylose, generated from residual *E. grandis* wood chips during pulp production, could be fermented into fuel, Silva et al. (2011) optimized ethanol production from hemicellulose hydrolyzate, generated from mild acid pretreatment. Dilute sulfuric acid was mixed with the wood chips and was then autoclaved (121°C, 45 min) to allow separation from the hemicellulose hydrolyzate portion from the solids’ (cellulose and lignin) portion. The hydrolyzate was then fermented to ethanol by a *Pichia stipitis* strain, known for its ability to ferment xylose, to achieve an ethanol concentration of 15.3 g/L (100 L/tonne of biomass). As a comparison, the solids’ portion, which was delignified using an alkaline NaOH pretreatment step (4%, w/v, 121°C, 20 min), was fermented by *S. cerevisiae* by an SSF process yielded a final ethanol concentration of 28.7 g/L.

Although this study demonstrates eucalypt biomass conversion from debarked biomass, bark accounts for approximately 10–12% of tree biomass residue processed from a plantation (Perlack et al., 2005; Zhu and Pan, 2010), which contains considerable levels of glucose (40%) and xylose (10%) (Lima et al., 2013). Given that bark is often not considered or optimized during lignocellulose pretreatment, Lima et al. (2013) tested various options for bark deconstruction from commercial *E. grandis* (EG) and *E. grandis* × *urophylla* (EGU) trees. The authors tested both one- and two-step acid and alkaline combinations in order to maximize sugar recovery. A combination of acid (1%) and NaOH (4%) pretreatment resulted in a solids fraction containing high concentrations of glucose from EG and EGU (78 and 81% dry weight, respectively); however, only 54.2 and 66.6% of total glucose was actually recovered after treatment. Upon saccharification, 65.4 and 84.5% of glucose was released from the acid + alkaline-treated bark samples. Alternatively, a single NaOH (4%) pretreatment step, while retaining lesser amounts of glucose within the solids fraction (56 and 62%), resulted in higher total recovered glucose (63.4 and 73.1%) and more efficient enzymatic saccharification (78.5 and 98.6%).

Although alkaline pretreatments are widely used, particularly in the pulp and paper industry, the chemicals required are considered pollutants and require multiple purification steps for removal from hydrolyzate. More recently, ILs, organic salts that are liquid at room temperature act as a solvent to solubilize cellulose, hemicellulose, and lignin without degradation, have been used as an effective pretreatment (Zhu et al., 2006). ILs, although not yet developed for large-scale use, are prized for their stability, recyclability, and low volatility during biomass solubilization (Zhu et al., 2006; Shi et al., 2015). As an emerging, pretreatment option, their exact interaction with biomass during solubilization is not well understood. To examine changes in cell wall structure and composition in woody biomass in response to IL pretreatment, Çetinkol et al. (2010) compared the cell wall of *E. globulus* before and after exposure to IL 1-ethyl-3-methyl imidazolium acetate [C2min][OAc]. Using a variety of imaging and spectroscopy techniques [2-dimensional nuclear magnetic resonance spectroscopy (2D-NMR), Fourier transform infrared spectroscopy, scanning electron microscopy, small angle neutron scattering, and X-ray diffraction], they found IL pretreatment resulted in the deacetylation of xylan, acetylation of lignin, and the selective removal of G lignin monomers thereby increasing the S/G ratio. Subsequent saccharification of the treated biomass showed a significant increase in glucose (5×) yield after 1 h saccharification, which authors attributed to a decrease in cellulose crystallinity. Xylose yield was also increased after IL treatment, which was undetectable after saccharification of untreated biomass.

Depending on their chemistry, ILs interact with biomass differently. Protic ILs (PILs) can be prepared via a one-step process with low-cost acids and bases and preferentially solubilize lignin, while aprotic IL (AIL) preparation is a multistep process and preferentially dissolve carbohydrate macromolecules (Greaves et al., 2006; Zhang et al., 2015). Zhang et al. (2015) developed a concerted IL pretreatment (CIL) for *Eucalyptus* bark, combining pyrrolidinium acetate ([Pyrr][AC]; PIL) with 1-butyl-3-methylimidazolium acetate ([BMIM][AC]; AIL). Compared to untreated bark, each IL pretreatment alone ([Pyrr] or [BMIM]) or separate combinations of each ([Pyrr] and [BMIM]), the CIL pretreatment ([Pyrr]/[BMIM]) resulted in 91% enzymatic hydrolysis of cellulose, as compared to 5, 67, 50, and 77%. The same trend (13, 48, 65, and 79%) was observed during enzymatic hemicellulose hydrolysis as well (untreated biomass, [Pyrr], [BMIM], [Pyrr] and [BMIM], and [Pyrr]/[BMIM]). Reduced lignin content correlated with cellulose conversion, which was further enhanced through the removal of hemicellulose. These strategies of converting underutilized (bark, thinned trees, and hemicellulose hydrolyzate) or undesirable (tension wood) lignocellulose will be a key for the sustainable generation of biofuels through coupling bioenergy production with traditional industrial forestry practices (van Heiningen, 2006).

While acid pretreatment remains a common method of pretreatment due to its effectiveness, strong industrial acids are expensive to generate and difficult to recycle and neutralize (Menon et al., 2010). An alternative pretreatment method utilizes residues on the xylan backbone to disrupt the structure of lignocellulose. Hot water pretreatment, or autohydrolysis, is a cost-effective pretreatment option that mixes pressurizes hot water with biomass in a reaction vessel, causing acetyl residues on the xylan backbone to generate *in situ* acetic acid. The internal generation of acetic acid reduces the pH of the biomass liquor and accelerates delignification and the solubilization of hemicellulose (Galbe and Zacchi, 2007). To demonstrate the effectiveness of liquid hot water pretreatment for eucalypt biomass, Yu et al. (2010) developed a two-step pretreatment assay (step 1: 180–200°C, 0–60 min and step 2: 180–240°C; 0, 20, 40, and 60 min) to achieve maximize xylose recovery and minimize cellulose degradation. Their results demonstrated that during the first pretreatment step, degradation of xylose to furfural increases linearly with reaction severity, a trend which continues during the second pretreatment step where furfural concentration increases between 180 and 200°C then seemingly decreases through the formation of other aldehyde products. During the second pretreatment step, furfural and 5-HMF production increased steadily over time at constant temperature (200°C), demonstrating that extended pretreatments are detrimental for recovery of monomeric sugars. Temperature had the greatest effect on the formation of inhibitory products, with authors finding that shorter reaction times and lower temperatures (180°C, 20 min; 200°C, 20 min) maximized sugar recovery (96.6%) and enzymatic digestion (81.5%).

Although autohydrolysis pretreatment can effectively solubilize hemicellulose, cellulose will remain in its recalcitrant, crystalline form after pretreatment. To reduce cellulose crystallinity in conjunction with autohydrolysis pretreatment, Inoue et al. (2008) used ball milling to improve saccharification yield from *Eucalyptus* biomass. The authors demonstrated that milling alone for short periods of time (20 min) could dramatically reduce cellulose crystallinity from 59.7 to 7.6%, although only 44.2% of sugars were captured after saccharification. To achieve higher rates of enzymatic saccharification from ball-milled biomass (86.2%), restrictively long milling times were required (120 min). To combat this, the authors combined a hot water pretreatment (160°C, 30 min) and ball milling (20 min) step to yield approximately 70% of total sugars with a low enzyme loading [4 filter paper units (FPU)/g substrate]. By comparison, the same yields were achieved by hot water pretreatment (160°C, 30 min) or ball milling (40 min) separately, each requiring 10× enzyme loading (40 FPU/g). This study demonstrates how combining methods can effectively reduce the severity of the pretreatment required to deconstruct biomass, which will lessen the formation of inhibitory products and the costs associated with enzymatic saccharification.

Traditionally, lignocellulosic biofuel production required separated process vessels where polysaccharide hydrolysis was carried out independently from microbial fermentation. Separate hydrolysis and fermentation (SHF) required additional processing and distilling steps to remove contaminants that prevent biofuel production (Olofsson et al., 2008). To improve biomass conversion efficiency and reduce fuel production costs, SSF processes generate liquid fuel from sugars as they are hydrolyzed from a polysaccharide. The advantages of SSF over SHF include the use of a single reactor for production to reduce capital costs, lower accumulation of sugars which bolsters saccharification rate and yield, and the presence of ethanol in the reaction vessel helps reduce microbial contamination (Krishna and Chowdary, 2000; Olofsson et al., 2008). To examine the efficiency of organosolv (in this case, ethanol and water) pretreatment with SSF processes, Yáñez-S et al. (2013) pretreated *E. globulus* biomass using an SSF process with various substrate loadings (10 and 15%, w/v), thermostable yeast concentrations (6 and 12 g/L), and enzyme loadings (as expressed as cellulase FPU/β-glucosidase IU [10/20, 20/40, and 30/60]). The authors found that the highest ethanol concentration (42 g/L) was obtained from 15% (w/v) substrate loading, 20 FPU/40 IU enzyme loading, at either yeast concentration. Although higher substrate loading decreased the overall ethanol yield, ethanol concentration within the reaction vessel was increased. Furthermore, mass balance calculation from 15% substrate loading within SSF and SHF processes suggested that greater ethanol amounts could be achieved by SSF (164 and 107 L/tonne, respectively).

The strategy of increasing the solids loading during an SSF is another strategy to further reduce operation costs associated with fuel production. By increasing the weight of solids to 15–20% of the SSF reaction, the energy required to heat and distil the reaction is dramatically reduced (Wang et al., 2011). Of course, this requires optimization of process parameters, such as liquid-to-solid ratio (LSR) and enzyme-to-substrate ratio (Romaní et al., 2011). Optimization of these parameters with *E. globulus* biomass, as well as autohydrolysis pretreatment severity, allowed Romaní et al. (2012) to reach an ethanol concentration of 67.4 g/L, representing 91% conversion of ethanol from cellulose, which scales to 291 L of ethanol per tonne of biomass.

Steam explosion (SE), another cost-effective pretreatment that is similar to autohydrolysis, solubilizes hemicellulose and disrupts the structure of biomass through the breakage of linkages caused by a sudden drop in pressure. SE pretreatment is often combined with alkaline or dilute acid catalysts to increase saccharification through either delignification or increased recovery of xylose (respectively). However, addition of catalysts increase biofuel production costs either through the cost of the chemical itself or through the additional washing and neutralization steps. Thus, optimization of SE pretreatment can provide an environmentally friendly process for biofuel production. Romaní et al. (2013) optimized the temperature (173–216) and pretreatment time ranges (6–34 min) with fixed enzyme loadings (15 FPU/10 IU) to improve ethanol production from *E. globulus* biomass. Using a scanning electron micrograph (SEM) to visualize the biomass after explosion, the authors observed that exposure to a temperature of 210°C for 30 min completely opened up the fibular structure of the biomass. Although, maximum ethanol production of the SE treated material was achieved under less severe conditions (210°C, 10 min) which produced 50.9 g/L from an SSF reactor. This represents again a 91% theoretical conversion of ethanol from cellulose, scaling to 248 L/tonne of biomass.

Microbial fermentation efficiency is another limiting step during lignocellulosic biofuel production. High-fuel production strains of yeast can readily convert glucose to ethanol while withstanding ethanol toxicity but are largely unable to utilize hemicellulose derived pentose sugars (Lange, 2007). Alternate strains, belonging to *Pichia* and *Candida* genera, are capable of xylose fermentation but lack productivity. Metabolic engineering achieved through transformation to generate an organism capable of efficiently utilizing multiple carbon sources will greatly increase lignocellulosic fuel production, particularly one unfettered by high concentrations of ethanol or aldehydes, such as furfural and 5-HMF (Sun and Cheng, 2002; Wen et al., 2009). Despite eucalypt’s desirable biofuel characteristics, their preferred climate ranges from cool temperate to tropical rainforest (Grattapaglia and Kirst, 2008; Shepherd et al., 2011). As such, their productivity as an energy crop outside of these climates is limited. To combat this limitation, Castro et al. (2014) investigated *E. benthamii*, a naturally cold resistant species that is commercially grown in Southeast USA, as a potential biofuel feedstock. To maximize biomass conversion, authors used a process known as liquefaction plus simultaneous saccharification and cofermentation (L + SScF), which combines dilute acid SE pretreatment with SSF processes with an inhibitor-resistant *E. coli* strain (SL100) capable of dual glucose/xylose fermentation. In addition, the authors used phosphoric acid instead of sulfuric acid, as it forms fewer inhibitory products during deconstruction and it allows the use of lower grades of stainless steel in reaction vessels, which saves on capital costs. Through optimization of temperature, acid concentration and pretreatment time (combined as a function of CSF), Castro et al. (2014) found that sugar yields were affected primarily by pretreatment time and temperature, with acid concentration having the smallest impact. Within the reaction vessel during fermentation, glucose was completely consumed within 48 h of fermentation, at which point the SL100 strain began fermenting xylose for the remainder of the 96 h fermentation. The cofermentation strategy to utilize all available carbon for conversion was successful, producing 240 g of ethanol/kg of raw biomass (304 L/tonne). For comparison, average ethanol production from sugarcane bagasse using the same process achieved 270–280 g/kg (342 SScF 355 L/tonne) (Geddes et al., 2013). Given the low costs of producing woody biomass (Hamelinck et al., 2005), this combination of strategies to employ alternative chemicals, SSF reaction vessels and cofermentation microbial strains that are engineered to withstand the detrimental effects of inhibitors demonstrates the feasibility of using eucalypts as a cost-effective crop for bioenergy production.

While ethanol is the most widely produced biofuel due to its ease of production, butanol is another fermentation product that can be used as a liquid fuel. Butanol is less volatile, hygroscopic, corrosive, and explosive than ethanol, can be transported with current infrastructure, and has similar energy content to gasoline (Antoni et al., 2007; Dürre, 2007; Ezeji et al., 2007; Fortman et al., 2008). Despite its advantages, microbial fermentation to butanol lacks efficiency given butanol’s toxicity and often requiring nutrient supplementation which increasing operating costs (Zheng et al., 2015). Zheng et al. (2015) demonstrated the feasibility of acetone–butanol–ethanol (ABE) production from *Clostridium saccharoperbutylacetonicum* from steam exploded *Eucalyptus* biomass without nutrient supplementation. Various glucose concentrations (30–75 g/L) were achieved though varying solid loadings (6.7–25%) finding that a hydrolyzate loading of 10% (39.5 g/L) generated the highest concentration of ABE (acetone 4.07 g/L, butanol 7.72 g/L, and ethanol 0.467 g/L). However, further optimization of glucose concentration (dilution of 75–45 g/L) produced the highest ABE concentrations (4.27 g/L acetone, 8.16 g/L butanol, and 0.643 g/L ethanol). Solids loading beyond 10% had a detrimental effect on ABE production, likely due to formation of fermentation inhibitors such as 5-HMF and phenolics.

## Lignin

Lignin, being the second most abundant biopolymer in plant tissue, accounts for roughly 25% of biomass. Its primary role is to provide strength and rigidity to the plant, as well as assisting in vascular water transport and protection from pathogens (Boerjan et al., 2003; Ralph et al., 2004). While providing critical functions for the plant, lignin effectively surrounds structural polysaccharides within the secondary cell well, resulting in inefficient release of fermentable sugars from chemical or enzymatic hydrolysis (Hinchee et al., 2010; Jönsson et al., 2013).

Lignin synthesis begins with the conversion of phenylalanine to trans-cinnamic acid, catalyzed by the enzyme phenylalanine ammonia lyase (PAL). The remaining enzymatic steps have been well-reviewed (Ona et al., 1997; Li et al., 2006; Déjardin et al., 2010; Vanholme et al., 2010), but ultimately this biosynthetic pathway ends with the generation of the main precursors of the lignin molecule: coniferyl, *p*-coumaryl, and sinapyl alcohol (Bonawitz and Chapple, 2010). Upon transportation to the secondary cell wall, each alcohol precursor undergoes an oxidation reaction, mediated by laccase and peroxidase enzymes, which destabilize the monolignol causing it to form a covalent bond with another monolignol. Once bonded, these subunits form ρ-hydroxyphenyl (H), guaiacyl (G), and syringyl (S) lignin (Ralph et al., 2004; Bonawitz and Chapple, 2010; Vanholme et al., 2010). The most common covalent bond to occur, particularly in eucalypt lignin, is the β-θ-4 linkage, which is predominately formed from S lignin monomers. Other linkages are present, such as β–β and β-5 dimers, but β-θ-4 linkages are preferential for pulp and biofuel production as they are less stable than other bonds, branch less frequently, and are more easily broken during alkaline pretreatment (Huntley et al., 2003; Hinchee et al., 2010).

Lignin represents the largest barrier to efficient deconstruction of woody biomass. Studies performed in transgenic lines of alfalfa, poplar and *Arabidopsis* have demonstrated how slight alterations in the quantity and composition of lignin can result in large downstream effects for the saccharification of biomass (Chen and Dixon, 2007; Leplé et al., 2007; Eudes et al., 2012). Given its importance to the survivability of the plant, genetic control of cell lignification is tightly regulated. Using the promoter region of cinnamoyl CoA reductase (*CCR*) from *E. gunnii*, paired with a reporter gene (*GUS*), Lacombe et al. (2000) demonstrated using transgenic tobacco plates that *EgCCR* was highly activated during development and lignification of xylem tissues. Control of the lignin biosynthetic pathway is achieved through AC-rich elements within gene promoters. These AC elements serve as a binding platform for transcription factors (such as LIM and MYB) that modulate gene expression (Rogers and Campbell, 2004; Zhong and Ye, 2007). The LIM transcription factor, first identified in tobacco, upregulates lignin genes. When silenced in tobacco using antisense *NtLIM1* constructs, transcripts for phenylpropanoid genes *PAL*, 4 coumarate CoA ligase (*4CL*), and cinnamyl alcohol dehydrogenase (*CAD*) were also downregulated, resulting in plants with 27% less lignin than wild type (Kawaoka et al., 2000). Similarly, suppression of the *LIM1* ortholog in *E. camaldulensis* also downregulated the *PAL*, *4CL*, and *CAD* gene pathways, resulting in plants with not only 29% less lignin but also 5% higher structural polysaccharides. The polysaccharide increase could be a result of shifting carbon resources as a result of downregulating the phenylpropanoid pathways (Kawaoka et al., 2006).

The MYB transcription factor, first discovered as a regulator of the lignin pathway in snapdragons, also affects the transcription of the lignin gene pathways. Identified from cDNA libraries of differentiating xylem tissue, the *E. grandis MYB2* gene when overexpressed in tobacco resulted in abnormal secondary cell wall thickening and altered lignin composition. Interestingly, while the expression of phenylpropanoid genes was unaltered, downstream genes responsible for monolignol synthesis [*4CL*, ρ-coumarate 3-hydroxylase (*C3H*), hydroxycinnamoyl:shikimate hydroxycinnamoyl transferase (*HCT*), caffeoyl CoA *O*-methyltransferase (*CCoAOMT*), ferulate 5-hydroxylase (*F5H*), caffeic acid *O*-methyltransferase (*COMT*), *CCR*, and *CAD*] were upregulated, increasing the S/G ratio composition of the lignin (Goicoechea et al., 2005). Another MYB transcription factor, identified from *E. grandis*, *EgMYB1*, when overexpressed in poplar and *Arabidopsis* resulted in plants with dwarfed leaves and stems and downregulated lignin and cellulose and hemicellulose transcripts. Given that the upregulation of *EgMYB1* resulted in the alteration of the major components of secondary cell wall structures suggests that MYB1 is a weak activator of lignocellulose genes, and its upregulation outcompetes stronger activators, thereby reducing overall transcription (Rogers and Campbell, 2004; Legay et al., 2010).

To investigate the effects of various wood properties on the enzymatic saccharification of woody biomass, such as lignin content, S/G ratio, cellulose crystallinity, fiber pore size, and enzyme adsorbtion, Santos et al. (2012) characterized the biomass of nine woody plants, including *E. nitens*, *E. globulus*, and *E. urograndis*. Using a Kraft alkaline pretreatment and fixed enzyme loading, the authors found that of all the parameters investigated, lignin content is the most significant contributing factor for saccharification. *E. globulus* biomass conversion resulted in the highest sugar recovery, efficient enzymatic conversion, and least residual lignin (75.2, 97.9, and 6.9%, respectively). However, lignin content alone did not fully explain saccharification yields, as biomass with similar lignin levels released much less glucose than *E. globulus*. Lignin S/G ratios were also found to impact enzymatic hydrolysis, as increased S lignin monomers undergo less frequent branching, producing a more linear polymer which increases enzymatic access to polysaccharides. Although, the effect of S/G ratio on saccharification appears to be dependent on biomass pretreatment, as acid hydrolysis has been shown to have a greater effect on low S/G lignin (Davison et al., 2006) while Papa et al. (2012) demonstrated using three mutant lines of *E. globulus* with varying S/G ratios (0.94, 1.13, and 2.15) that lignin composition did not affect saccharification after IL pretreatment.

Given that lignin remains the largest barrier to effective deconstruction of woody biomass for fermentation, treatments to increase the efficiency at which it can be removed from biomass will aid biofuel production. To improve enzymatic saccharification of eucalypt biomass, Sykes et al. (2015) generated transgenic *E. grandis* × *urophylla* hybrids with RNA interference (RNAi)-downregulated lignin biosynthetic genes *C3H* and cinnamate 4-hydroxylase (*C4H*). Total lignin content in transgenic lines was reduced by 8–9%, and after hot water pretreatment (designed as a mild, cost-effective method for biomass disruption) and enzymatic saccharification, both *C3H* (94%) and *C4H* (97%) transgenic lines released higher total sugars than control biomass (80% saccharification). However, transgenic lines were dwarfed (*C3H* – 2.0 m and *C4H* – 3.4 m) as compared to controls (6.0 m), a common issue for lignin transgenic plants that could be alleviated through silviculture practices.

Until low lignin transgenic plants are further developed, large-scale biofuel production will depend on harsher pretreatments that inhibit microbial growth and enzymatic action through solubilization of phenolics (Ximenes et al., 2010; Jönsson et al., 2013). An alternative option to aid in delignification of biomass is the addition of laccase to destabilize the lignin network through phenol oxidation. Gutiérrez et al. (2012) and Rico et al. (2014) tested the potential of a laccase enzyme to increase saccharification from *E. globulus* biomass. Tested in the presence of an enzyme mediator, either 1-hydroxybenzotriazole (HBT) or methyl syringate (respectively), both studies reported lignin reduction (~48%) in *E. globulus* substrate and increased glucose and xylose yields after saccharification. Using pyrolysis-gas chromatography/mass spectroscopy to understand the effect of the laccase treatment, authors found an increased S/G composition (4.9 vs. 4.0) within the lignin because of preferential hydrolysis of G lignin subunits, resulting in a less condensed phenolic polymer. Continued investigation of laccase pretreatment with mediators was conducted by Rico et al. (2015) using 2D-NMR to characterize each step of delignification by fungal enzymes with *E. globulus* biomass and cellulolytic lignin. The low redox potential *M. thermophila* laccase enzyme and methyl syringate mediator pretreatment was tested against a high redox potential laccase, isolated from *Pycnoporus cinnabarinus*, with HBT mediator across several stages of pretreatment and alkaline extraction. Though various structural changes occurred throughout each stage of the fungal pretreatments, the most striking effects involved the preferential removal of guaiacyl units, reduced β-0-4 alkyl–aryl ether linkages, and S/G ratio increase. Syringyl lignin subunits underwent C_α_ oxidation during laccase pretreatment, which were incompletely removed through alkaline extraction. Both fungal enzyme treatments achieved similar delignification results (~50%), although multistage analysis suggests that the rate of oxidation by *P. cinnabarinus* laccase + HBT was greater. The 50% delignification result correlated with a 30% increase in glucose yield after enzymatic saccharification. These results suggest that the largest gains in sugar release from biomass result from total delignification of biomass rather than the alteration of lignin composition.

While *M. thermophila* is a commercially available strain, its laccase enzymes may lack specificity when applied to various lignocellulose feedstocks. To investigate novel laccase enzymes from endophytic fungi, occurring in symbiosis with *Eucalyptus* trees, Martín-Sampedro et al. (2015) screened more than 100 strains, selecting five for their ligninolytic enzymes. These strains, tested against a white rot *Trametes* sp. reference, were combined with 10 g of *Eucalyptus* wood chips, before or after mild autohydrolysis pretreatment (selected to minimize the production of fungal inhibitory products). Enzymatic saccharification of each pretreatment released greater sugar yields from combination of treatments (fungal + autohydrolysis) than either pretreatment alone. Endophytic fungi strains *Ulocladium* sp. and *Hormonema* sp. outperformed the *Trametes* sp. reference strain, resulting in 3.3- and 2.9-fold increase of total sugars (compared to a 2.3-fold increase) as compared to autohydrolyzed control biomass (~3 g/L). The authors postulated that the specific activity of the ligninolytic enzymes could be a result of evolutionary processes, and endophytic fungi represent a large reservoir of biodiversity to aid biofuel production.

## Conclusion

Given the global demand and potential for lignocellulosic biofuels, selection and research into alternative feedstocks is essential. Eucalypts, given their wide range of phenotypic diversity, genetic potential, environmental adaptability, and desirable cell wall chemistry, are excellent candidates for bioenergy production (Table 2). While eucalypt biomass is highly prized for other industrial processes, such as pulp and paper and timber production, the most economical way to introduce lignocellulose into the energy supply chain will be in conjunction with other plantation practices where thinned and undesirable trees are removed to promote growth of high-value trees. In addition, the production of fuel from waste wood chips and bark within pulping factories will help convert mills into complete biorefineries. Indeed, as global paper consumption diminishes, alternative uses for eucalypt biomass will require research and development. While pulping plants are efficient at deconstruction, harsh pretreatments are not suitable for downstream microbial conversion of polysaccharides to monosaccharides to fuel. High temperatures and pressures, while effective for deconstruction, generate inhibitory compounds from lignin and carbohydrates that result in sugar losses and inefficient downstream processes. Lignocellulosic fuel will require mild, low-cost pretreatments, coupled with SSF or “one-pot” processes to promote efficient biofuel production.

**Table 2:** **Advances in lignocellulosic biofuel production from eucalypt biomass**.

Reference	Strategy	Pretreatment and fermentation conditions	Conclusions	Result
Inoue et al. (2008)	Pretreatments without acids/bases/solvents are cheaper with fewer environmental impacts	Autohydrolysis + milling	Duel pretreatment required 10× less enzyme for saccharification	70% sugar recovery
Yu et al. (2010)	Two-step liquid hot water hydrolysis of biomass	Autohydrolysis	Temperature affects degradation products formation	96.6% sugar recovery; 81.5% saccharification
			Short reaction times and low temperatures maximize recovery	
Çetinkol et al. (2010)	IL pretreatment of biomass	1-Ethyl-3-methyl imidazolium acetate	Deacetylation of xylan	5× glucose yield
			Acetylation of lignin	
			Increased S/G ratio	
Silva et al. (2011)	Hemicellulose deconstruction and fermentation from residual wood chips	Dilute sulfuric acid	Hemicellulose was separated from cellulose and lignin	15.3 g/L ethanol (100 L/tonne biomass)
		*P. stipitis* (*S. cerevisiae* fermentation of solids)		28.7 g/L ethanol (obtained from solids)
Muñoz et al. (2011)	Fermentation of tension and opposite wood	Organosolv	Tension wood required milder conditions to delignify	35 g/L ethanol (290 L/tonne biomass)
		SSF fermentation with *S. cerevisiae*		
McIntosh et al. (2012)	Optimization of acid concentration, temperature, and pretreatment time	Sulfuric acid	Hemicellulose solubilizes and degrades first	18 g/L ethanol
		*S. cerevisiae*	Temperature contributes most to glucose release	
Santos et al. (2012)	Screened various woody feedstocks with varying for wood properties	Alkaline pretreatment	Lignin content, enzyme adsorbtion, and S/G ratio contribute most saccharification	*E. globulus* biomass (low lignin content 7%, 98% saccharification, and 75% sugar recovery)
Papa et al. (2012)	Investigate effects of S/G ratio on IL pretreatment efficiency	1-Ethyl-3-methyl imidazolium acetate	S/G ratio did not affect IL pretreatment efficiency	Glucose yield of 759–897 g/kg cellulose after 24 h saccharification
Yáñez-S et al. (2013)	SSF optimization of substrate loading, yeast concentration, and enzyme loading	Organosolv	Higher substrate loading and midrange enzyme loading maximize yield	42 g/L ethanol (164 L/tonne of biomass)
Romaní et al. (2012)	SSF optimization of substrate and enzyme loading	Autohydrolysis and SSF reaction	91% conversion of cellulose to ethanol	67.4 g/L ethanol (291 L/tonne of biomass)
Romaní et al. (2013)	Optimization of temperature and pretreatment time	Steam explosion and SSF reaction	Maximum ethanol yield is achieved at 210°C for 10 min	50.9 g/L ethanol (248 L/tonne of biomass)
Lima et al. (2013)	Optimization of pretreatment for *Eucalyptus* bark	One/two-step acid/alkaline pretreatment	Single alkaline step recovered most glucose	73.1% glucose recovery and 98.6% saccharification
Castro et al. (2014)	SSF fermentation with inhibitor-resistant cofermentation *E. coli* strain	Steam explosion + phosphoric acid	Sugar yield is primarily determined by pretreatment time and temperature	240 g ethanol/kg biomass (304 L/tonne biomass)
		SSF fermentation + cofermentation *E. coli*		
Rico et al. (2014, 2015)	Fungal laccases with mediator pretreatment	Laccase pretreatment + alkaline extraction	Preferential G unit removal	~50% lignin reduction and 30% increase in saccharification
			S unit oxidation	
			Increased S/G ratio	
Martín-Sampedro et al. (2015)	Screening, isolation, and pretreatment with endophytic fungal laccases	Fungal pretreatment + autohydrolysis	Endophytic fungi outperformed white rot reference *Trametes* strain	3.3 and 2.9× increase in total sugar release after pretreatment
Sykes et al. (2015)	RNAi downregulation of lignin genes *C3H* and *C4H*	Hot water pretreatment	Transgenic lines had less lignin and underwent more efficient saccharification	*C3H* (94% saccharification)
			Transgenic plants were dwarfed	*C4H* (97% saccharification)
				Control (80% saccharification)
Zhang et al. (2015)	IL pretreatment of eucalyptus bark	Pyrrolidinium acetate and 1-butyl-3-methylimidazolium acetate	IL combinations had a synergistic effect on pretreatment	91% enzymatic hydrolysis of cellulose
Zheng et al. (2015)	ABE production without nutrients	Steam explosion and *Clostridium* fermentation	Solids loading and glucose concentration are critical for microbial inhibition	4.27 g/L acetone, 8.16 g/L butanol, and 0.643 g/L ethanol

*IL, ionic liquid; S, syringyl; G, guaiacyl; SSF, simultaneous saccharification fermentation; RNAi, RNA interference; C3H, ρ-coumarate 3-hydroxylase; C4H, cinnamate 4-hydroxylase; ABE, acetone/butanol/ethanol*.

Genetic and chemical exploitation of eucalypt cell walls has allowed the design of mild and environmentally friendly pretreatments, such as autohydrolysis and SE, relying on *in situ* acid generation to aid deconstruction without expensive and caustic chemicals. Although these pretreatments help to reduce the formation of inhibitory products, aldehydes and phenolics formed from cellulose, hemicellulose, and lignin will likely remain in low concentrations within reaction vessels, necessitating the need for robust fermentive microbial strains. Metabolic engineering to exploit genetic variation has great potential to overcome the largest barriers to fuel conversion. These techniques have already generated dual fermentation stains to utilize all present carbon sources and resist the effects of degradation products within reaction vessels to main productivity. Application of the same principles to feedstocks have downregulated lignin gene pathways, designing plant cell walls that deconstruct with ease under mild conditions. Coupled with screening and isolation of endophytic fungi with specific ligninolytic enzymes, lignin deconstruction and removal from process vessels will maximize enzyme adsorbtion, sugar recovery, and fermentation.

Ionic liquids are the most promising for biomass pretreatment, given their stability and low volatility, and action at low temperatures. Despite the commercial use of cold resistant *E. benthamii*, eucalypts are not the ideal biofuel feedstock in all climates. ILs work universally well regardless of feedstock composition, solubilizing whole biomass without degradation, and selectively precipitating cellulose upon the addition of an antisolvent. Efficient saccharification of the cellulose precipitate maximizes sugar recovery and maintains intact lignin for alternate chemical processing.

In addition to the biological components of biofuel production, process optimization, such as single reaction SSF and high solids’ loading, increases achievable ethanol concentrations and lower capital costs for production. Additional savings will be gained through the combination of pretreatments to reduce energy costs and enzyme loading for efficient saccharification. Increased understanding of eucalypt cell wall formation, particularly lignin formation, will allow the engineering of new and effective pretreatment options to make biofuel production suitable for a wide range of lignocellulosic feedstocks to provide renewable fuels for the future.

## Conflict of Interest Statement

The authors declare that the research was conducted in the absence of any commercial or financial relationships that could be construed as a potential conflict of interest.
